# 

**Published:** 1892-04

**Authors:** 


### Communications.

AMERICAN ACADEMY OF DENTAL SCIENCE.—At a meeting of the American Academy of Dental Science, held in Boston, March 2, 1892, the following resolutions on the death of Dr. C. A. Kingsbury were adopted.

WHEREAS Charles A. Kingsbury, M.D., D.D.S., late of Philadelphia, an associate member of the American Academy of Dental Science, has recently been called away from these earthly scenes into the higher existence beyond the grave, we, his fellow members, would offer the following tribute of respect to his memory.

*Resolved*, That in the death of Dr. Kingsbury, the profession has lost one of its brightest and most honored members, and the city and community in which he lived, one of its most exemplary and useful citizens. Dr. Kingsbury was engaged in active practice and professor of dental histology and operative dentistry in the Philadelphia Dental College through a long series of years, and contributed much to elevate and adorn his profession. He lived to an advanced age, and had the pleasant satisfaction in his declining years of seeing the science and art of dentistry, and the standing of the profession, far advanced towards the ideal that he had formed for it in the thoughts and aspirations of his earlier years.

*Resolved*, That we tender our sympathy and condolence to his surviving family in their heavy bereavement and sorrow, and the Corresponding Secretary be requested to forward a copy of these resolutions to them, and that a copy be entered upon the records of the Academy, and published in the dental journals.

C. A. BRACKETT, D.M.D.,
*President.*

E. N. HARRIS, D.D.S., *Cor. Sec.*

---

AMERICAN DENTAL SOCIETY OF EUROPE.—The American Dental Society of Europe will hold its eighteenth meeting at Basle, Switzerland, August 1st, 2nd, and 3nd. Members of the profession are cordially invited to attend. Clinics will be a special feature of this meeting. The University will place desirable rooms at the disposal of the Society, and an ingenious amphitheater for accommodating in the immediate vicinity of the patient a larger number of spectators than are able to witness operations under the ordinary circumstances will be loaned by the Swiss Dental Association. Programmes may be had on application to the President, Dr. Bryan of Basle, or to Chas. W. Jenkins, Secretary.

---

KENTUCKY STATE DENTAL ASSOCIATION.—The twenty-second annual meeting will be held at Louisville, Ky., Tuesday, Wednesday and Thursday, June 21, 22 and 23, 1892, at the Louisville College of Dentistry, Chestnut street, between Floyd and Preston.

J. H. BALDWIN, D.D.S.,
*Secretary.*

---

THE annual meeting of the National Association of Dental Examiners will be held at Niagara Falls, Monday, August 1st, 1892, at ten (10) A. M.

All State Boards are invited.

FRED. A. LEVY, *Sec'y.*

---

### Monthly Summary.

### To Readers and Correspondents.

Communications intended for publication, and exchange journals and papers, should be addressed to the Editor of The American Journal of Dental Science, No. 845 N. Eutaw St. Baltimore, Md. All letters relating to the business of the Journal, or containing cash remittances, to be directed to Messrs. Snowden & Cowman, No. 9 W. Fayette Street, Baltimore, Md. Articles for publication should be received by the Editor at least two weeks previous to the first of the month on which the number in which it is designed for them to appear, is issued.

☞ The American Journal of Dental Science will contain fifty pages of reading matter in each number, making a volume of about Six Hundred pages,

EXCLUSIVE OF ADVERTISEMENTS

